# The effect of five years versus two years of specialised assertive intervention for first episode psychosis - OPUS II: study protocol for a randomized controlled trial

**DOI:** 10.1186/1745-6215-12-72

**Published:** 2011-03-10

**Authors:** Marianne Melau, Pia Jeppesen, Anne Thorup, Mette Bertelsen, Lone Petersen, Christian Gluud, Gertrud Krarup, Merete Nordentoft

**Affiliations:** 1 Psychiatric Centre Copenhagen, Copenhagen University, Faculty of Health Sciences, Copenhagen, Denmark; 2 Copenhagen Trial Unit, Centre for Clinical Intervention Research, Rigshospitalet, Copenhagen University Hospital, Copenhagen, Denmark; 3 Psychiatric University Hospital Risskov, University of Aarhus, Faculty of Health Sciences, Aarhus, Denmark

## Abstract

**Background:**

The Danish OPUS I trial randomized 547 patients with first-episode psychosis to a two-year early-specialised assertive treatment programme (OPUS) versus standard treatment. The two years OPUS treatment had significant positive effects on psychotic and negative symptoms, secondary substance abuse, treatment adherence, lower dosage of antipsychotic medication, and a higher treatment satisfaction. However, three years after end of the OPUS treatment, the positive clinical effects were not sustained, except that OPUS-treated patients were significantly less likely to be institutionalised compared with standard-treated patients. The major objective of the OPUS II trial is to evaluate the effects of five years of OPUS treatment versus two years of OPUS treatment.

**Methods:**

The OPUS II trial is designed as a randomized, open label, parallel group trial with blinded outcome assessment. Based on our sample size estimation, 400 patients treated in OPUS for two years will be randomized to further three years of OPUS treatment versus standard treatment. The specialized assertive OPUS treatment consists of three core elements: assertive community treatment, psycho-educational family treatment, and social skills training.

**Discussion:**

It has been hypothesized that there is a critical period from onset up to five years, which represents a window of opportunity where a long-term course can be influenced. Extending the specialized assertive OPUS treatment up to five years may allow the beneficial effects to continue beyond the high-risk period, through consolidation of improved social and functional outcome.

**Trial registration:**

Clinical Trial.gov NCT00914238

## Background

The yearly incidence of patients aged 18 to 35 years with schizophrenia in Denmark in 2008 was approximately 800. The yearly incidence for patients of the same age with other disorders in the schizophrenia spectrum (F 21 - F 29, ICD -10[1]) was almost 500. Schizophrenia is a severe neurodevelopmental disorder with complex neuronal and psychosocial pathogenesis. The first psychotic break down is usually seen in adolescence or early adulthood and has a serious impact on young peoples' lives through interference with their social lives and work. Furthermore, patients with psychosis often suffer from substance abuse, depression [2], suicide [3], and are often associated with high rates of violence and legal problems[4]. The direct cost of schizophrenia in European countries has been estimated to two percent of the national health expenditures[5], - a similar order of magnitude to cancer or ischaemic heart disease. Moreover, there are also huge indirect costs to society in terms of suffering of relatives and lost productivity[5].

The focus on first-episode psychosis arises because converging evidence suggest that the underlying illness process that affects biological, psychological, and social domains undergo major deterioration around the onset phase of the illness[6-8]. Delayed detection and treatment is a widespread problem and predicts poor clinical outcome [9,8]. The association between longer periods of untreated psychosis and poorer outcomes is firmly established[8,9]. It is therefore of utmost importance to identify possibilities for prevention and treatment. The evidence base for the psychosocial treatment of psychotic disorders is still underdeveloped[10]. Historically, randomized clinical trials have been few in number and uneven in quality[11]. There is an urgent need for large investigator-initiated, independent, non-commercial trials of complex interventions that can help us prevent the invalidating and deteriorating course in many patients.

The Danish OPUS I trial randomized 547 patients with first-episode psychosis to a two-year specialised intensive assertive treatment programme (OPUS) consisting of a multimodal phase-specific treatment of first episode psychosis versus standard treatment[12]. The results significantly favoured the OPUS treatment[12], showing positive effects on psychotic and negative symptoms, secondary substance abuse, treatment adherence, lower dosage of antipsychotic medication, and a higher treatment satisfaction after two years of treatment. However, the five-years follow up study, three years after patients from OPUS were transferred to standard treatment, showed that most of the positive clinical effects were not sustained[13]. This concurs with the British LEO trial[14,12], demonstrating that specialised treatment for people with first-episode psychosis is effective on psychotic and negative symptoms only as long as the treatment continues.

It has been hypothesised that there is a critical period up to five years after onset of psychotic illness, which represents a window of opportunity where the long-term course can be influenced[6]. The objective of the present OPUS-II trial is therefore to carry out a comparison of five-years versus two-years of specialised assertive intervention for first episode psychosis. Our hypothesis is that extending the specialised assertive intervention service up to five years will allow the beneficial effects to continue beyond this high-risk period, through consolidation of improved social and functional outcomes. The trial will thereby provide clinicians and planners of mental health services with the needed evidence regarding the optimal duration of specialised treatment of first-episode psychosis patients. The cost-benefit of a specialised assertive intervention sustained for the entire critical period is equally important to establish.

## Methods

### Design

**Figure 1:**
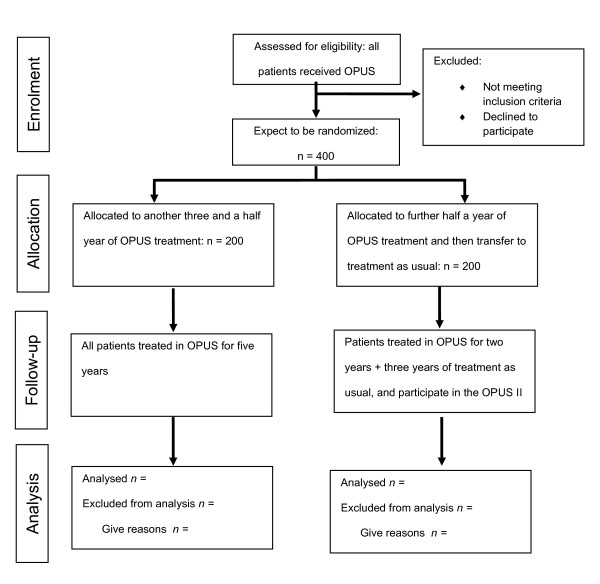
**CONSORT diagram depicting flow of study participants in the OPUS II trial**.

The study is designed as a randomized, open label, parallel-group trial with blinded outcome assessment (Figure 1). Given the nature of the question, patients and health care providers cannot be kept blind to treatment allocation. There are five OPUS-teams in the Capital Region and one OPUS-team in Central Denmark Region.

### Participants

All patients treated within the Danish mental health services for at least 11/2 year by the six OPUS teams will be approached for eligibility. Patients must fulfil the ICD 10 diagnostic criteria for schizophrenia or schizophrenia-like psychosis (F2)[1].

#### Inclusion criteria

patients aged 18-37 years with first episode psychosis in schizophrenia spectrum, who in the inclusion period of the trials are treated for at least 11/2 year by one of the six OPUS teams will be asked to give signed informed consent to participate in the trial.

#### Exclusion criteria

patients whose diagnosis was evaluated retrospectively and found not to fulfil the criteria for first-episode psychosis in the F2-spectrum (ICD 10) at any time during the first 11/2 year of OPUS treatment, and patients who do not give signed informed consent to participate in the trial. Drug and alcohol misuse will not lead to exclusion. Patients who are mentally retarded in moderate to severe degree are not treated in OPUS teams and are therefore not a part of the inclusions and exclusions criteria.

Randomization will take place approximately six months prior to the termination of the original two-year OPUS treatment. This will allow a gentle transfer to other treatment services for the control group, and allow proper time to plan the future course of treatment for the experimental group. Expected duration of participating in the trial is therefore 3 1/2 year from entry interview to follow-up interview.

### Randomisation

Randomisation will be centralised and computerised with concealed randomisation sequence carried out by the Copenhagen Trial Unit (CTU). Block size will be unknown to the investigators and clinicians. Randomisation will be stratified for treatment site (six sites) and severity of global score on the four of the five domains in SANS, anhedonia, avolition, alogia and affective blunting, with distinction between at least one of the global score measured less than 3 compared to 3 or more. Signed informed consent will be obtained prior to patients being randomized. The allocations concealment is ensured by the investigators call to the randomisation unit, CTU, after completing the collection of baseline data and data needed for the randomisation.

### Interventions

The trial is pragmatic, comparing five years versus two years of specialised, assertive intervention programme (OPUS) defined by a set of protocols.

#### OPUS treatment

The integrated OPUS-treatment consists of three core elements: assertive community treatment, psycho educational family treatment, and social skills training. In addition, the patients receive group intervention to facilitate recovery, cognitive behaviour therapy when indicated, and crisis intervention. All patients are designated a primary staff member, who are responsible for maintaining the contact and to coordinate the treatment within the team, but also coordinate across social services and other institutions involved in the treatment. The pharmacological treatments are guided by official Danish guidelines[15]. The team offers psycho-educational family treatment, and will always try to get in contact with at least one family member and motivate the family to participate in a psycho-educational group. Patients will be visited in their homes or other places in their community or at their primary team member's office according to the patient's preferences. OPUS-treatment is tailored to meet the individual patient's needs.

The OPUS staff consists of a multidisciplinary team, including a psychiatrist, psychologists, nurses, social workers, physiotherapist, and vocational therapist. All team members, except the psychiatrist, function as primary team member for the patients. The patient to staff member ratio is 10:1. All members of the OPUS staff are well educated, and have obtained great experience in first episode psychosis, and are continually trained and supervised in the core elements of the OPUS treatment to provide the specialised assertive intervention.

#### The OPUS treatment in the experimental extension period (year 2-5)

The aim is to continue the original patients - primary team member relation in the extension period, if possible. Patient to staff member ratio is 15:1. Patients will still be offered treatment tailored to meet their individual needs. As a minimum the patients will receive at least one face-to-face contact each month with their primary team member, and at least one other contact (e.g., telephone, mail). Families can in the extension period still join the psycho-educational family treatment due to their needs, and will at minimum be invited to booster sessions of a survival skills workshop. Patients can participate in all group programmes available in OPUS. Within the resources of the team, there is no upper limit for the amount of treatment the individual patient may obtain.

The intensity of intervention will be registered. Interventions intensity will be extracted from the Danish Psychiatric Case Register. In case of doubt, data will be cross-validated with data extracted from the patient's medical record. Cost of intervention will be assessed with help from the economists in regional health authorities.

#### Standard treatment (year 2-5)

Patients, who after 11/2 years of OPUS treatment, are randomized to further half a year of the specialised assertive intervention, will be transferred to standard treatment after a total of two years OPUS treatment. Standard treatment can be either affiliation to a community mental health centre, assertive community treatment, or primary care, depending on the patient's needs. The most common will be referral to community mental health care centres, which usually offer the patient treatment in which home visits are possible but office visits are the general rule. The patient to staff member ratio varies between 20:1 and 30:1.

The transition from the OPUS treatment to standard treatment will be carried out gradually and as gentle as possible.

### Monitoring programme fidelity

Monitoring the fidelity of the intervention to OPUS treatment will be carried out by an independent investigator interviewing the six OPUS team leaders, using the index of fidelity to Assertive Community Treatment (IFACT)[16]. Assertive community treatment (ACT) and the OPUS treatment have some key elements in common. However, the OPUS interventions are enhanced by more specific content aimed at patients with first episode psychosis, including better family involvement and social skills training. To address these core elements we will develop a programme fidelity measure with special registration of presence or absence of critical components in the OPUS treatment. These elements are: primary contact to one member of the team, psycho-education, crisis plan, social skills training, rehabilitation support, psycho educational family treatment, and multifamily group.

### Outcome measures

The primary outcome measure is: negative symptoms measured with schedule for assessment of negative symptoms in schizophrenia (SANS[17]). Investigators at entry and at the 31/2 years follow-up interview will be independent and blinded for treatment allocation. Through training interviews, we will ensure that the assessors have a high level of interrater reliability with kappa values for primary outcome measure of at least 0.70, prior to beginning the trial. We will also ensure that assessors have an interclass correlation coefficient (ICC) of at least 0.70 regarding SANS global scores. ICC is calculated in SPSS using two-way mixed models for absolute agreement on single measures.

The secondary outcome measures are: simultaneously remission of both negative and psychotic symptoms, measured with SANS and schedule for assessment of positive symptoms in schizophrenia (SAPS[17]), psychotic symptoms measured as continuous measures, substance abuse, working alliance, self efficacy, user satisfaction, adherence to treatment, compliance with medication, suicidal behaviour, use of bed days, ability to live independently, and labour market affiliation. The criteria for remission are that none of the global measures of severity in SAPS (global scale for hallucinations, delusions, bizarre behaviour, or thought disorder) and SANS (global scale for anhedonia, avolition, alogia, and affective blunting) exceeds a value of 2 (2 = mild symptoms). All outcome measures are measured with validated scales (Table 1). These outcomes will be assessed at baseline and at follow-up. Through training interviews, we will also ensure that assessors have an ICC coefficient of at least 0.70 regarding SAPS global scores.

**Table 1:** Assessment instruments in the OPUS-II trial.

Topic:	Instrument:
Psychopathology	Schedules for Clinical Assessment in Neuropsychiatry (SCAN)[28], Schedule for assessment of positive and negative symptoms in schizophrenia (SAPS and SANS[17])[29], Hamilton's depressions scale[30], Suicidal behaviour, DUP, Duration of Untreated Psychosis[31]

Social function	Personal and Social Performance scale, PSP[32] Global Assessment of Functioning, GAF[33]

Cognitive function	Brief Assessment of Cognition in Schizophrenia, BACS [34]

Medication and side effect	The UKU side effects rating scale, UKU[35] Patients self rapport

Selfefficacy	General Self - efficacy Scale[36]

Alliance	Working Alliance Inventory, client, WAI[37]

Quality of life	WHO Quality of Life, QOL[38]

Client satisfaction	Client Satisfaction Questionnaire (CSQ)[39]

Vital status, cause of death [18], use of beddays[19]	Danish national registers

Socio demographic information, labour market affiliation, civil status, cohabitation status, children, education, living in an institution for mentally ill, living in an institution for homeless[20]	Danish national registers

To be able to evaluate the external validity of the trial, we will furthermore register sex, age, diagnosis, medical treatment, substance abuse, and treatment adherence for the patients who do not participate in the trial.

#### Register-based information

vital status, cause of death [18], use of mental health services[19], living in an institution for mentally ill, living in an institution for homeless, labour market affiliation, sick leave, and early age pension will be extracted from the unique, complete, longitudinal Danish registers[20]. Use of antipsychotic medication will be extracted from the Danish Register of Medicinal Product Statistic[21].

#### Participant withdrawal

participants can withdraw his/hers informed consent from the trial at any time without any explanation. We have obtained permission from the Danish Data Protection Agency (journal number: 2009-41-3314) for register-based information for all randomized patients, and in analyses of this information, we will include data from all patients.

### Sample size calculation

In the OPUS I trial we found that the patients treated in OPUS had a mean score of 1.42 in negative dimension and patients treated in standard treatment had a mean score of 1.84 in negative dimension with standard deviation of 1.2[22,12]. In this present OPUS II trial we want to be able to detect a difference of 0.4 point in negative dimension, measured with SANS. We are thus planning a trial of a continuous response variable from independent control and experimental participants with one control per experimental participant. If the true difference in the experimental and control means is 0.4 with a standard deviation of 1.2, we will need to study 142 experimental participants and 142 control participants to be able to reject the null hypothesis that the population means of the experimental and control groups are equal with probability (power) 0.80. The type I error probability associated with this test of this null hypothesis is 0.05, and 200 patients will be included in each intervention group because we expect approximately 30% attrition.

### Data analyses

The primary outcome measure, negative symptoms, will, as other continuous outcome measures, be subjected to analysis using a mixed-model analysis with a repeated measurements model with unstructured variance matrix, using the mixed-model command in SPSS. This approach assumes that the distribution of missing data can be estimated from the information from previous interviews and from information about other patients in the database. The condition for using this method is the assumption that data are missing at random or missing completely at random when taking into consideration the information extracted from baseline interviews and information about the other patients in the database. In this model, baseline values of the scales are included. Variables included as covariates will be site (six different teams), sex, co-morbid substance abuse, baseline values of the remission variable, and the baseline values of variables that differ significantly in drop-out analyses. Besides the above-mentioned variables, compliance with medication, level of school education, and whether or not a family member is involved in treatment will be included in analyses of differences between patients that are lost to follow-up and those who remain in the trial. If significant, these variables will be included as covariates.

For evaluation of the dichotomous secondary outcome measure (simultaneously remission of psychotic and negative symptoms or not), we will use multiple multivariate imputations, using all other covariates to impute a distribution of missing values. The dichotomous outcomes will be analysed with logistic regression analyses. Variables included as covariates will be the same as those included in analyses of continuous measures. Similar to the repeated measurement, the condition for using multiple imputations is that data are missing at random or missing completely at random, when taking into consideration the information extracted from baseline interviews and information about the other patients in the database.

Analysis will be based on the intention-to-treat principle. Data from all patients will be included in the group to which random assignment is made, regardless of intervention received.

### Feasibility

We plan to recruit 400 patients for the trial from 2009 to 2011. This is realistic as each of the six OPUS teams discharges approximately 40 to 50 patients per year.

### Ethical considerations

The result of this trial can be of great importance for future health-care planning to benefit people with a first-episode psychosis. The extended treatment period will, as mentioned earlier, be tailored to meet the patient's individual needs, witch means monitoring the treatment intensity up and down to support the patient's empowerment and recovery process. The design of the trial does not put participants at any unacceptable level of risk and the trial design uses the best form of standard care as the control group intervention.

### Informed consent

All potential participants considered for this trial will be provided with written and oral information on this trial so that they can make an informed decision about their participation. The participants must sign this consent form before being randomized. This protocol was submitted the Regional Ethics Committees for The Capital Region for review (journal no H-C-2009-035). The Committee assessed the protocol to be exempt from formal approval with the reason that the trial is a non-biomedical trial. For further detail information please contact: http://www.cvk.sum.dk

The participants will be offered written information about the findings and the results after analysing the OPUS II trial. The information is offered in form of a standard letter and will be posted to those who sign up for this opportunity.

The OPUS II trial has permission from the Danish Data Protecting Agency: J. no. 2009-41-3314 and is publicly registered (Clinical Trial Gov Identifier no.: NCT 00914238).

## Discussion

The strengths of the OPUS II trial is the high internal and external validity[23]. The use of a computer generated random sequence generation handled by an external partner (the Copenhagen Trial Unit) reduces the risk of selection bias. Secondly, the use of blinded outcome assessors for the primary outcome and the use of intention-to-treat analysis aim to prevent biased effect estimate[23].

The publication of the current design article and registration of the trial at 'clinical.trials.gov' are intended to prevent selective outcome reporting. The risk of spurious findings, random error, is reduced, in case the pre-specified sample size is reached.

The fact that we are not able to blind the participants and personnel in the extension period of OPUS treatment might increase the risk of performance bias. Due to the novelty and excitement of participating in a research project it could be argued, that both participants and staff conducting the experimental intervention would be more enthusiastic and keen to perform well and thereby offer a higher level of OPUS treatment than the actual intervention itself.

All potential patients in the catchments area are approached and offered OPUS II treatment and together with few exclusion criteria it provides a high level of external validity. However, the educational requirements for OPUS staff are a potential limitation to less developed health-care services.

Negative symptoms as measured with SANS are the primary outcome measure for this trial. Negative symptoms are associated to the patients experience of quality of life and the level of social functions[24,25], and therefore is likely to have a great impact on peoples live through interference with their social and educational lives. Furthermore, negative symptoms have been shown to have higher prognostic value than psychotic symptoms[26,27].

A further strength of our trial is our aim to monitor program fidelity and to address the presence or absence of critical components in the OPUS treatment. Currently, no single accepted validated standard to measure the program fidelity of first episode psychosocial treatment exists, and we hope to be able to contribute to the establishment of such a standard.

## Abbreviations

ACT: Assertive Community Treatment; CTU: Copenhagen Trial Unit; CSQ: Client Satisfaction Questionnaire; DUP: Duration of Untreated Psychosis; GAF: Global Assessment of Functioning; ICC: Interclass Correlation Coefficient; IFAC T: Index of Fidelity of Assertive Community Treatment; OPUS: is not an abbreviations, OPUS is a name borrowed from the world of music; PSP: Personal and Social Performance scale; QOL: Quality Of Life; SANS: Schedule for Assessment of Negative symptoms in Schizophrenia; SAPS: Schedule for Assessment of Positive symptoms in Schizophrenia; SCAN: Schedules for Clinical Assessment in Neuropsychiatry; UKU: Side effect rating scale; WAI: Working Alliance Inventory;

## International collaboration

The OPUS II research group has a close collaboration with Professor Ashok Malla, Douglas Hospital Research Centre (McGill University), Principal Investigator of the trial 'A randomized controlled evaluation of extended specialized early intervention service vs. regular care for management of early psychosis over five year critical period'. We will ensure that the OPUS II trial and the Canadian trial are comparable regarding intervention, assessments tools, and other methodological questions ensuring that the results can be meaningfully meta-analysed.

## Competing interests

The authors declare that they have no competing interests.

## Authors' contributions

M.Sc. Marianne Melau, Dr. Gertrud Krarup, and Prof. Merete Nordentoft are responsible for inclusion of patients and research interview. Dr. Anne Thorup, Dr. Lone Petersen, Dr Pia Jeppesen, Psychologist, Dr Mette Bertelsen, and Dr. Christian Gluud were involved in planning of the trial, critical comments to the manuscript, and approval of the final version of the manuscript.
